# Training-Induced Acute Neuromuscular Responses to Military Specific Test during a Six-Month Military Operation

**DOI:** 10.3390/ijerph18010215

**Published:** 2020-12-30

**Authors:** Kai Pihlainen, Arto J Pesola, Joonas Helén, Keijo Häkkinen, Taija Finni, Tommi Ojanen, Jani P. Vaara, Matti Santtila, Jani Raitanen, Heikki Kyröläinen

**Affiliations:** 1 Training Division, Defence Command, P.O. Box 919, 00131 Helsinki, Finland; 2 Active Life Lab, South-Eastern Finland University of Applied Sciences, P.O. Box 68, 50101 Mikkeli, Finland; arto.pesola@xamk.fi; 3 Department of Military Pedagogy and Leadership, National Defence University, P.O. Box 7, 00861 Helsinki, Finland; joonas.helen@mil.fi (J.H.); jani.vaara@mil.fi (J.P.V.); matti.santtila@kolumbus.fi (M.S.); heikki.kyrolainen@jyu.fi (H.K.); 4 Neuromuscular Research Center, Faculty of Sport and Health Sciences, University of Jyväskylä, P.O. Box 35 (VIV), 40014 Jyväskylä, Finland; Keijo.Hakkinen@jyu.fi (K.H.); taija.m.juutinen@jyu.fi (T.F.); 5 Human Performance Division, Finnish Defence Research Agency, P.O. Box 5, 04401 Järvenpää, Finland; tommi.ojanen@mil.fi; 6 UKK Institute for Health Promotion Research, P.O. Box 30, 33501 Tampere, Finland; jani.raitanen@ukkinstituutti.fi; 7 Faculty of Social Sciences (Health Sciences), Tampere University, P.O. Box 100, 33014 Tampere, Finland

**Keywords:** readiness, physical fitness, soldier, occupational performance, electromyography

## Abstract

Limited data are available regarding strength and endurance training adaptations to occupational physical performance during deployment. This study assessed acute training-induced changes in neuromuscular (electromyography; EMG) and metabolic (blood lactate, BLa) responses during a high-intensity military simulation test (MST), performed in the beginning (PRE) and at the end (POST) of a six-month crisis-management operation. MST time shortened (145 ± 21 vs. 129 ± 16 s, −10 ± 7%, *p* < 0.001) during the operation. Normalized muscle activity increased from PRE to POST in the hamstring muscles by 87 ± 146% (116 ± 52 vs. 195 ± 139%EMG_MVC_, *p* < 0.001) and in the quadriceps by 54 ± 81% (26 ± 8 vs. 40 ± 20%EMG_MVC_, *p* < 0.001). In addition, higher acute BLa values were measured after MST during POST. Changes in BLa and EMG suggested an increased neural input and metabolic rate during POST MST, likely leading to faster performance times at the end of the operation. High EMG values throughout the different phases of MST suggested that despite the anaerobic nature of the test, the soldiers were able to maintain their voluntary muscle activation level until the end of the test. This indicates only limited neural fatigue during the two-minute high-intensity military specific performance. While learning effect may explain some part of the improvement in the MST performance times, combined strength and endurance training three times per week may improve neuromuscular performance in occupationally relevant tasks.

## 1. Introduction

Several duties performed during military operations such as patrolling in combat gear and material handling tasks are physically demanding [1,2]. Furthermore, maintenance of physical performance may be challenged during deployments due to increased environmental and operative demands, mental and physical stress, as well as limited access and time to train [3,4]. In fact, several studies have reported decreases in aerobic fitness after prolonged (>nine months) military deployments [5,6,7,8]. In addition, neuromuscular performance may be attenuated within a few weeks of detraining [9].

Deterioration of physical fitness may have a negative impact on occupational performance and combat readiness during sustained military operations. Loss in physical fitness of an individual would increase the relative physiological demands of performing a task and reduce overall working capacity during prolonged assignments [10], which in many occasions, is the nature of military duties. Thus, while individual modifications to external (e.g., task originated) occupational demands may often not be possible during military operations, one strategy for decreasing relative physical workload includes improvement of physical fitness [11,12].

Changes in physical performance of soldiers can be assessed by traditional fitness tests, but more task specificity may be achieved by using occupationally relevant simulations. A high-intensity military simulation test (MST) lasting on average two minutes was developed for crisis management soldiers to test their physical performance in a possible combat situation during patrolling [13]. In combination with other physical performance measures, this cross-sectional study reported that the power of the lower extremities, endurance performance, muscle mass, and muscular endurance of the upper extremities were relevant fitness predictors, explaining two thirds of the variance in the MST performance time [13]. However, these cross-sectional results do not provide information about training adaptations during deployment and thus, follow-up tests are required for longitudinal assessment. In addition, changes in acute neuromuscular and metabolic variables provide more in-depth knowledge about the mechanisms behind the chronic training adaptations. Regarding to the study of Pihlainen et al. [13], high blood lactate (BLa) and rating of perceived exertion (RPE) levels after MST indicated that the performance induced increased glycolytic metabolism and acute subjective fatigue. It was reported that when oxidative metabolism is not sufficient to provide energy for the required exercise intensity level, increased rates of glycolysis result in enhanced lactic acid production in muscles and accumulation of blood lactate, which in turn may lead to symptoms of fatigue [14].

Nowadays, commercial skin surface electromyography (sEMG) garments can be utilized for the measurement of duration and intensity of dynamic neuromuscular function during occupational settings [15,16,17]. Despite the sEMG technology development, assessment of muscle activity has rarely been utilized in applied military field studies, but more so in standardized laboratory conditions [18,19,20]. For example, Paul et al. [19] observed a 242% increase in muscle activity of the knee extensors in soldiers carrying a 21.4-kg military specific load on a treadmill with a pace of 4 km/h and 25% gradient, when compared to the 0% gradient condition. Thus, the main purpose of the present study was to assess acute training-induced changes in the MST performance, with special focus on muscle activity and metabolic rates, during a six-month military operation.

## 2. Materials and Methods

A longitudinal follow-up design during a 6-month crisis management operation was employed to compare training-induced changes in muscle activity during the MST in soldiers. Baseline measures (PRE) were collected within ten days after a two-week non-standardized acclimatization phase at the deployment area. Twenty weeks later, the measurements were repeated (POST) in the same order three weeks before returning to homeland from deployment. Strength training frequency and volume load (upper body, lower body), as well as endurance training frequency and volume within three heart rate (HR) -based intensity zones (<75% HR_peak_, 75–85% HR_peak_, >85% HR_peak_) were recorded between PRE and POST by using training diaries.

Forty-nine voluntary male soldiers (age [mean ± standard deviation] 28[±8] years, height 180[±6] cm, body mass 79[±8] kg) with a minimum military training experience of 9 months, who were deployed to a crisis management operation in the Middle East for 6 to 12 months took part in the present study. The exclusion criteria for participation in the crisis management operation included health limitations with a need of permanent medication and endurance performance lower than 2300 m in the 12-min running test [21]. The study was approved by and conducted in accordance to the statement of the Ethics Board of the Central Finland Health Care District (KSSHP E1/2013). The soldiers were informed of the benefits and risks of the investigation prior to signing an institutionally approved informed consent to voluntarily participate in the study. The total number of soldiers participating the PRE and POST MST measurements was 81 and 69, respectively. However, while soldiers with both PRE and POST MST, MVC and EMG measurements were included in the data, the final number of participants was 49 soldiers. With the exception of one voluntary withdrawal from the study, the loss of data was due to military duties that prevented the soldiers from participating in the measurements.

The assessments of strength, endurance, and military task-specific performance were performed on separate days with a minimum of 24 h between the tests [13]. Anthropometric measurements were conducted in the morning after an overnight fast at a military hospital. Body mass and body composition variables—skeletal muscle mass, fat mass, and fat percentage—were assessed using segmental multi-frequency bioimpedance analysis (InBody 720, Biospace Co Ltd., Seoul, Korea).

Bilateral maximal isometric force (MVC) of the extensor muscles of the lower extremities was measured in a sitting position (knee angle 107°, hip angle 110°) using an electromechanical dynamometer [22]. Explosive force production of the lower extremities, dynamic muscle endurance capacity of the trunk, and upper extremities were assessed using standing long jump (SLJ) [23], 1-min sit-up [24], 1-min push-up [25], and pull-up tests, respectively. A supervisor demonstrated correct techniques before each test. For SLJ, the best out of three attempts, defined as the longest distance from the starting line to the landing point, was selected for further analysis. For the sit-up and push-up tests, the results were expressed as the number of consecutive repetitions in 60 s. Finally, the result of the pull-up test was expressed as the number of consecutive repetitions until volitional exhaustion. Endurance performance was assessed using the 3000-m running test (3000-m) time as the outcome measure. HR_peak_ was determined for endurance training prescription by a computer analysis software (Firsbeat PRO, Firstbeat Technologies, Jyväskylä, Finland) as the highest recorded HR during 3000-m.

**Figure 1:**
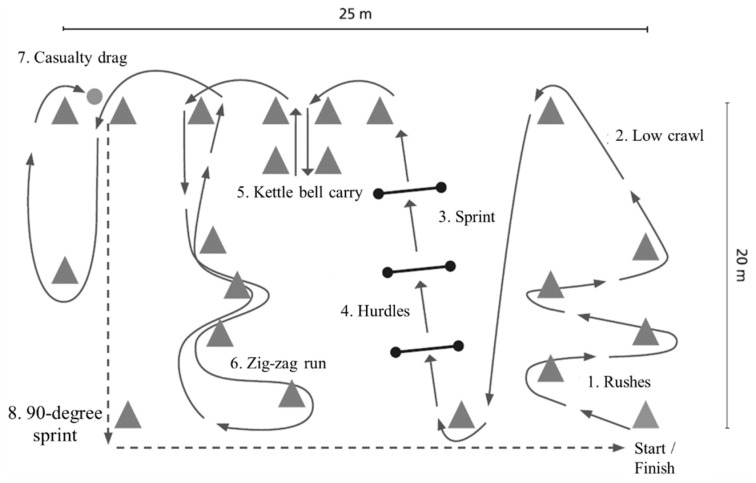
Illustration of the military simulation test track, including eight tasks (1–8) of the test.

Military occupational performance was assessed by MST [13], which consisted of typical army soldier maneuvers and tasks (Figure 1). The test was performed wearing sEMG shorts, leather boots, combat uniform and gear including body armor, helmet (total weight 19 ± 1 kg), and an assault rifle replica (3 kg). The test consisted of short rushes with changes in direction, low-crawling, sprints with and without hurdle jumps, manual material handling, and casualty evacuation. A standardized warm-up/practice trial was performed on the track before the test trial. The total length of the track was 242.5 m and the outcome measure was the duration of the test performance. BLa and, Borg’s [26] rating of perceived exertion (RPE) using the scale of 6 (no fatigue) to 20 (maximal fatigue), were additionally recorded before and after MST.

sEMG of the quadriceps femoris (QF) and hamstrings (HM) muscles was recorded during MVC using large textile electrodes embedded inside compression-type shorts allowing for minimal sensor movement (Myontec Mbody sizes S-XL, Kuopio, Finland) in order to determine one second average maximal EMG value during MVC (EMG_MVC_). Similarly, sEMG was recorded throughout the entire MST. The data processing method has been described in more detailed by Tikkanen et al. [15]. Briefly, the sEMG data was recorded to a small module containing signal amplifiers, microprocessor, and a computer interface. The raw data was recorded at a 1000-Hz sampling frequency (filtering bandwidth of 40–500 Hz/−3bD), rectified and averaged over 40 ms non-overlapping windows throughout the MST. Thereafter, the EMG data were downloaded to computer and artefacts were visually checked and removed in MegaWin software (Mega Electronics Ltd., Kuopio, Finland) following the procedures reported previously [15]. Signals from the left and right QF, as well as left and right HM muscles, were averaged to provide overall measures of QF and HM activities, respectively. For MVC, the EMG data were presented as absolute mean values (μV). For MST, the data were normalized to the EMG_MVC_ value over 1 s window, when the signal was greatest, averaged across the channels and reported as %EMG_MVC_ for the QF and HM muscles.

In addition to analyzing the whole MST, the eight tasks of MST (Figure 1) were separated from each other by concurrently utilizing video analysis and visual comparison of the EMG activities. The eight tasks were: rushes (1), low crawl (2), sprint (3), hurdles (4), kettle bell carry (5), zig-zag run (6), casualty drag (7), and a 90-degree sprint (8).

Sample size calculations were conducted to estimate the power of this sample to detect a meaningful change during the 6-month operation. Assuming an alpha error of 5%, a sample size of 49 gave 93% power to detect a medium effect (Cohen’s d = 0.5) change. The observed power was even higher (99%) due to the effect size for the change in MST over time (145 s to 129 s) being 0.64. Significance of changes in the measured variables were tested using the Student’s paired t-test (*p_s_*) or Wilcoxon signed rank test (*p_w_*), and *p* < 0.05 was used to establish statistical significance. Descriptive statistical methods were used for the calculation of means and standard deviations (SD). Using the relative change of the MST time as dependent variable, correlations and relationships with the respective changes in the normalized EMG, body composition and physical performance were investigated using Pearson moment correlation coefficient. Outliers (z-score < −3.3 or > 3.3) were detected and removed separately in each analysis. Commercial software (IBM SPSS 25.0.0.1, Chicago, IL, USA) was utilized for statistical analyses.

## 3. Results

**Figure 2:**
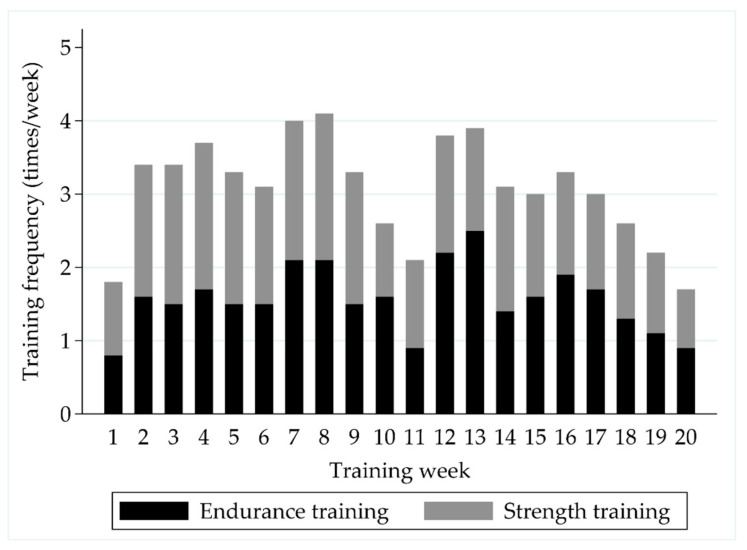
The distribution of self-reported weekly strength and endurance training sessions during the follow-up.

**Figure 3:**
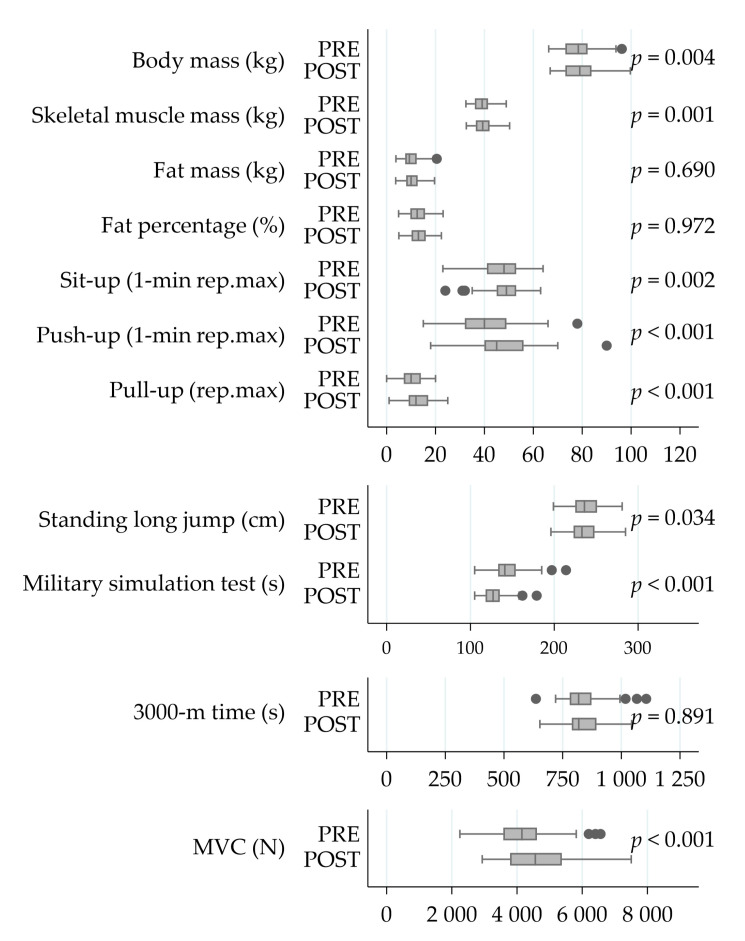
The changes in body composition and physical performance during the operation. Significances have been calculated using the Student’s paired-test. Abbreviations: PRE, at the beginning of a six-month crisis-management operation; POST, at the end of a six-month crisis-management operation; Rep.max, repetition maximum; 3000-m, 3000 m running test; MVC, bilateral maximal isometric force of the lower extremities.

The soldiers performed on average 1.7 ± 0.8 strength training sessions and 1.5 ± 1.2 endurance training sessions per week during the 6-month crisis management operation (Figure 2). The MVC of the leg extensor muscles increased by 11% (4215 ± 956 N vs. 4618 ± 1038 N, *p_s_* < 0.001) during the deployment (Figure 3). Also, the PRE-POST mean EMG amplitude of QF increased by 10% in the MVC measurement (753 ± 221 μV vs. 825 ± 267 μV, *p_w_* = 0.019), while no change (223 ± 115 μV vs. 273 ± 215 μV, *p_w_* = 0.312) was observed in HM.

**Figure 4:**
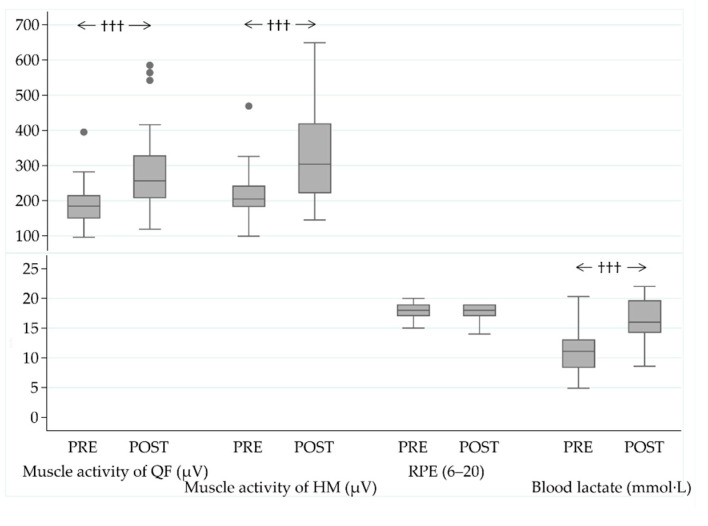
Muscle activity of the quadriceps femoris (QF) and hamstring (HM) muscles, acute responses in ratings of perceived exertion (RPE), and blood lactate (BLa) values after the military simulation test (MST) at the baseline (PRE) and in the end (POST) of the military operation. ††† *p* < 0.001 (Wilcoxon signed rank test).

The MST performance was improved by 10% (145 ± 21 vs. 129 ± 16 s, *p_s_* < 0.001) during the operation. The test resulted in higher absolute muscle activity values and acute BLa responses in the POST-measurement phase compared to the baseline, while RPE (18 ± 1 vs. 18 ± 1, *p_w_* = 0.70), assessed immediately after the MST, remained unchanged (Figure 4).

**Figure 5:**
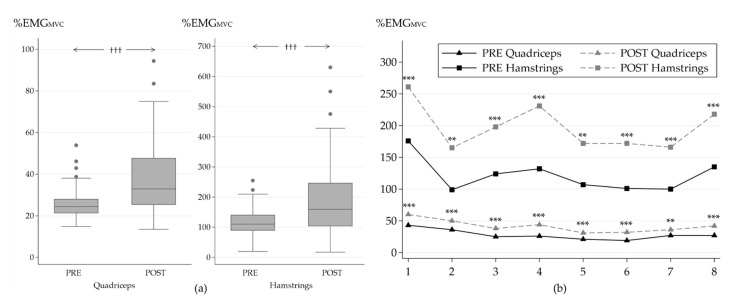
Average normalized muscle activity of the quadriceps and hamstrings muscles during MST (**a**) and during the individual tasks of MST (**b**) in the beginning (PRE) and at the end (POST) of the study. Significance of change between PRE and POST: ††† *p* ≤ 0.001 (Wilcoxon signed rank test); ** *p* ≤ 0.01; *** *p* ≤ 0.001 (Student´s paired t-test). MST tasks: 1, rushes; 2, low crawl; 3, sprint; 4, hurdles; 5, kettle bell carry; 6, zig zag run; 7, casualty drag; 8, 90-degree sprint.

Muscle activity increased in both of the muscle groups during the POST MST (Figure 5a). Normalized muscle activity increased from PRE to POST in HM by 87% (116 ± 52 vs. 195 ± 139%EMG_MVC_, *p_w_* < 0.001) and in QF by 54% (26 ± 8 vs. 40 ± 20%EMG_MVC_, *p_w_* < 0.001). Increases were also observed in all of the partitional tasks of the MST (Figure 5b). The highest relative increases were observed in task 4 (hurdles, 76 ± 135%, *p_s_* < 0.001) and task 6 (zig zag run, 78 ± 139%, *p_s_* < 0.001) for QF, while for HM, the highest relative increases were observed in tasks 2 (low crawl, 117 ± 256%, *p_s_* = 0.001) and 4 (hurdles, 107 ± 167%, *p_s_* < 0.001).

**Figure 6:**
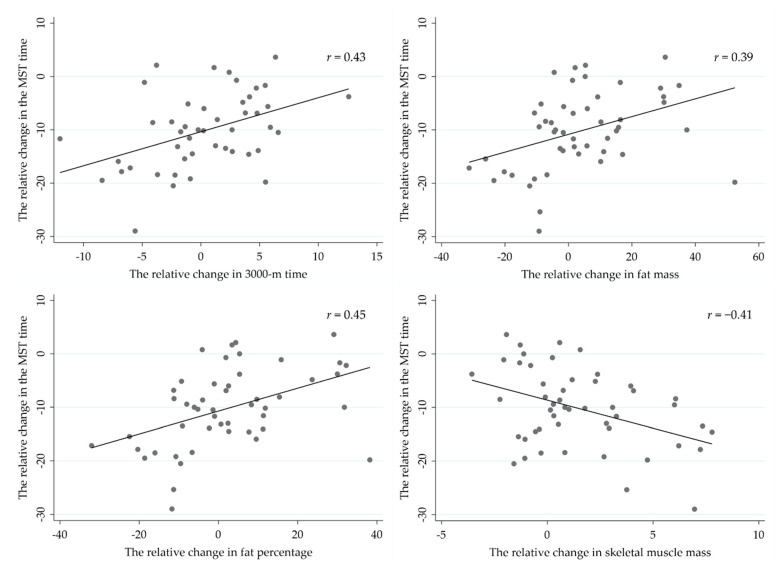
Pearson correlations between the relative change in military simulation test (MST) and respective changes in body composition and the 3000-m running test.

The strongest correlations between the relative change in MST time and respective changes in body composition and physical performance variables during the operation (Figure 6) included 3000-m time (r = 0.43, *p* = 0.003), fat mass (r = 0.39, *p* = 0.006), fat percentage (r = 0.45, *p* = 0.001) and skeletal muscle mass (r = −0.41, *p* = 0.004). In addition, negative correlations were found between relative change in MST time and number of push-ups in one minute (r = −0.29, *p* = 0.040), as well as between MVC force and MST time (r = −0.32, *p* = 0.026).

Correlative analyses did not reveal relationships between strength training variables and changes in muscle activity during MVC or MST. However, the increase in relative contribution of HM, namely QF/HM ratio during the MVC, correlated with higher volume of low-intensity endurance training (r = −0.57, *p* = 0.001), as well as with the volume of moderate-intensity endurance training (r = −0.41, *p* = 0.047). In addition, the volume of low-intensity endurance training correlated with the relative change in muscle activity of HM during the MVC (r = −0.58, *p* = 0.001).

## 4. Discussion

The present study demonstrated that combined strength and endurance training during the military operation increased muscle activation in the QF and HM muscles in the POST measurement phase and, at least partly, explained faster MST performance times. On the other hand, moderate correlations between longitudinal changes in the MST time and the respective changes in muscle mass, endurance running performance, and upper extremity strength endurance suggest that the MST performance may also have been enhanced by co-activation of synergist (e.g., upper extremities, torso) muscles that were not measured during the present study. In addition, some uncontrolled variables such as, most likely learning effect and/or pacing may have influenced performance times.

During voluntary muscle work, force output and coordination of agonist-antagonist and synergist muscles are regulated by recruitment and synchronization of motor units [27]. During fatiguing submaximal muscle work, motor unit excitation increases gradually, leading to activation of higher threshold motor units until the entire motor unit pool is activated and, thereafter, reduction in muscle activation, force production, and thus, physical performance, occurs [27]. For example, Muddle et al. [28] observed recruitment of larger motor units and increased firing rates, especially in higher threshold motor units during low-intensity (30% MVC) isometric exercise to failure, lasting approximately six and a half minutes. Compared to high-intensity (70% MVC) work-matched exercise lasting one and a half minute, recruitment threshold of larger motor units decreased more during lower intensity exercise, with the simultaneous increase in the threshold of smaller motor units. In both conditions, however, the overall EMG amplitude of the vastus lateralis muscle increased until the volitional failure [28]. The present study assessed dynamic muscle actions that varied within the tasks of MST, which makes the interpretation of data more complicated. However, the main finding was increased absolute and relative QF and HM muscle activity during the MST in the POST measurement phase. Thus, combined strength and endurance training performed on average, three times per week during the deployment, likely increased the activation of the thigh muscles, and resulted in improved occupational performance of soldiers, assessed with MST. This would be expected, since our prior study showed that 66% of the variance in the MST time was explained by explosive strength of the lower extremities, muscle mass, endurance running performance, and muscle endurance of the upper extremity extensor muscles [13]. These performance outcomes can be developed concurrently without interference if the training volume is modest [29], as in the present study.

Cross-sectionally, there was no clear trend in EMG amplitude change (e.g., increase or decrease) within the partitional tasks of the MST, showing that despite the anaerobic nature of the test, the soldiers were able to maintain their voluntary muscle activation level until the end of the test. For example, when muscle activities of similar tasks (Figure 5b) were compared between the early (e.g., task 3, sprint) and the last phase (e.g., task 8, final sprint) of the MST, only a slight increase in muscle activity was observed. Thus, it appears that neuromuscular fatigue did not limit the performance of soldiers during the test, even though their high BLa and RPE values after the test indicated high metabolic demand and subjective fatigue.

Izquierdo et al. [30] studied acute changes in BLa and EMG of the QF muscles during fatiguing resistance training protocol (5 × 10RM leg-press, adjusted independently for pre and post 1RM) before and after a 7-week strength training program. While maximal strength increased as a response to strength training program, the relative 5 × 10RM training induced similar EMG responses compared to baseline, but higher accumulation of BLa. They concluded that after the training program, higher absolute muscle work was performed at the same relative activation level, resulting in higher accumulation of metabolites [30]. As in the study of Izquierdo et al. [30], higher muscle activity as well as higher peripheral fatigue were observed during the POST measurements in the present study. It has also been shown that increased BLa level is associated with improved excitability of muscles during submaximal fatiguing exercise and that this effect is more pronounced in trained versus untrained subjects [31]. It needs to be emphasized that in the present study, the effort of soldiers was maximal, aiming to complete MST within the shortest possible time, while in the two abovementioned studies [30,31], the participants were performing the same relative submaximal work intensity until volitional exhaustion.

While significant increases in the absolute and relative QF and HM muscle activity were observed during the follow-up, muscle activity increased more in the HM muscles. This suggested that the soldiers improved utilization of the muscles involved with knee flexion and hip extension, when the HM muscles are working also as agonists, which is important in military tasks including rushes, sprints, and hurdle jumps. The importance of muscle strength of the hip extensors has been highlighted in studies focusing on load carriage performance [32] as well as lower extremity injury prevention in high intensity sports [33]. Even though linear relationships were not found between MST and strength or endurance training variables, higher low- and moderate-intensity endurance training volume was associated with increased HM activity during the MVC measurement.

Strengths and limitations of the present study should also be discussed. As far as the authors are aware of, there are no previous studies reporting acute neuromuscular responses, nor longitudinal adaptations to occupational military simulation during deployment. However, the quality of the study, as well as interpretation of the results would have improved, if a control group had been applied and thus, lack of it can be considered as a limitation of the study. Traditionally, EMG measurements have been conducted in laboratory settings and using small bipolar electrodes, aiming to measure activity of a certain motor unit. This is challenging and requires precise determinations of measurement sites, especially, in longitudinal field studies. Even though the electrodes were carefully placed on the exact same anatomical positions, there were several pitfalls affecting the signal outcome such as conductivity issues related to possible changes in subcutaneous fat content, or amplitude cancellation, caused by differences in timing of action potentials, etc. [34,35]. During dynamic muscle contractions such as performing MST, both force output and body postures change and thus, there existed more confounding factors for the EMG analysis. For example, electrode shift during or between the measurements may lead to recording of different muscle fibers with different recruitment thresholds [34]. To avoid these pitfalls, EMG amplitude was normalized to the respective MVC measurement. Furthermore, multiple electrodes, providing a more representative measure of muscle activity from a group of muscles, instead of a single motor unit, were used. As presented, this study used large area sEMG electrodes, which enabled quantification of the overall muscle activity of the quadriceps and hamstring muscle groups. The validity and reliability of textile EMG measurement method has been tested against traditional laboratory-quality skin surface electrodes during static and dynamic activity [36,37].

During the isometric leg press-type of the MVC measurement, motor units of QF were mainly responsible for total force output, while HM, supporting joint stability, only partly contributed to force production, and primarily as antagonist muscles in the leg press action. This method led to high (>100%) relative activation values of HM during the MST (Figure 5). The most optimal method of normalization would have been muscle (or muscle group) specific MVC with muscles of interest working as agonists, but this was not possible due to logistic limitations. While the present method was a standardized one, it may be regarded as one of the limitations of the study. Therefore, the present normalized MST values provide more comparable results of the role of QF, rather than HM. From this perspective, it may be stated that performance at the relative QF activation level of 30–40% EMG_MVC_ throughout the 2–3 min MST required high metabolic and subjective demand. More detailed research of fatigue requires EMG power spectrum methods to differentiate recruitment of motor units with different thresholds from changes in firing rates of active motor units, as both of them result in changes in EMG [38]. Also, spectral analyses require stationary electrode positioning to enable recording of the same motor unit pool, which may be very challenging during functional tests [34].

## 5. Conclusions

In conclusion, increased lower extremity muscle activity in the MST performance was observed during the six-month military operation, likely as a result of training-induced increase in physical fitness and motor learning. The assessment of muscle activity provided new insights into the physiology of the MST, revealing high neuromuscular input, which was in line with POST BLa and RPE values. In addition to military research, other physically demanding occupations (e.g., firefighting and law enforcement) could benefit from EMG field measurements in the assessment of muscle group dynamics, as well as limitations and effectiveness or training adaptations of the neuromuscular system [18,39].

## Data Availability

Data sharing is not applicable.
